# Analysis of Dental Specialty Center target achievement and associated factors, Brazil, 2019-2022: a cross-sectional study

**DOI:** 10.1590/S2237-96222025v34e20240610.en

**Published:** 2025-07-11

**Authors:** Luciana Leônia Soares Freire, Rênnis Oliveira da Silva, Rilary Rodrigues Feitosa, Lucas Xavier Bezerra de Menezes, Renato Taqueo Placeres Ishigame, Yuri Wanderley Cavalcanti, Edson Hilan Gomes de Lucena

**Affiliations:** 1 Universidade Federal da Paraíba, Programa de Pós-Graduação em Odontologia, João Pessoa, PB, Brazil; 2 Universidade Federal da Paraíba, Curso de Odontologia, João Pessoa, PB, Brazil; 3 Ministério da Saúde, Coordenação-Geral de Saúde Bucal, Brasília, DF, Brazil; 4 Universidade Federal da Paraíba, Departamento de Clínica e Odontologia Social, João Pessoa, PB, Brazil

**Keywords:** Oral Health, Secondary Care, Specialties, Dental, COVID-19, Health Evaluation, Salud Bucal, Atención Secundaria de Salud, Especialidades Odontológicas, COVID-19, Evaluación en Salud

## Abstract

**Objective:**

To evaluate the performance of Dental Specialty Centers in Brazil from 2019 to 2022 and analyze its association with contextual factors.

**Methods:**

This is an observational, cross-sectional, descriptive, and analytical study. We used secondary data on Dental Specialty Center production collected from the Outpatient Information Systems. We used a robust Poisson multiple regression model to determine the target achievement prevalence ratio (PR). We calculated 95% confidence intervals (95%CI) for the variables under study.

**Results:**

Poor performance, with achievement of 0-3 targets, prevailed in the Dental Specialty Centers. Among the dental procedures, targets related to general basic procedures had the best result, with the highest achievement of targets. Dental Specialty Centers located in regions with more than 100,000 inhabitants had a higher frequency of target achievement (PR 1.68; 95%CI 1.26; 2.29). In 2020 (PR 0.10; 95%CI 0.07; 0.15), 2021 (PR 0.22; 95%CI 0.16; 0.28) and 2022 (PR 0.48; 95%CI 0.39; 0.59) target achievement prevalence was lower than in 2019.

**Conclusion:**

Most Dental Specialty Centers performed poorly in achieving their targets. Municipalities with larger population sizes were more likely to achieve their targets. The COVID-19 pandemic negatively influenced their performance.

## Resumen

**Objetivo:**

Evaluar el desempeño de los Centros de Especialidades Odontológicas en Brasil, de 2019 a 2022, y analizar su asociación con factores contextuales.

**Métodos:**

Se trata de un estudio observacional, transversal, descriptivo y analítico. Se utilizaron datos secundarios sobre la producción de los Centros de Especialidades Odontológicas recolectados de los Sistemas de Información de Ambulatorios. Se obtuvo un modelo de regresión múltiple de Poisson robusto para determinar la razón de prevalencia (RP) de logro de metas. Se obtuvo el intervalo de confianza del 95% (IC95%) para las variables en estudio.

**Resultados:**

En los Centros de Especialidades Odontológicas prevaleció un desempeño pobre, con 0 a 3 metas alcanzadas. Entre los procedimientos, las metas relacionadas con procedimientos básicos generales tuvieron el mejor resultado, con el mayor cumplimiento de metas. Los Centros de Especialidades Odontológicas ubicados en regiones con más de 100.000 habitantes mostraron una mayor frecuencia de logro de metas (RP 1,68; IC95% 1,26; 2,29). Los años 2020 (RP 0,10; IC95% 0,07; 0,15), 2021 (RP 0,22; IC95% 0,16; 0,28) y 2022 (RP 0,48; IC95% 0,39; 0,59) mostraron una menor prevalencia de logro de metas que el año 2019.

**Conclusión:**

La mayoría de los Centros de Especialidades Odontológicas mostraron un desempeño deficiente en el logro de metas. Los municipios con mayor población tenían más probabilidades de alcanzar los objetivos. La pandemia de COVID-19 influyó negativamente en el desempeño.

**Ethical aspects**

This research used public domain anonymized databases.:

## Introduction

The Dental Specialty Centers are responsible for providing specialized oral health services, forming an important component of the Oral Health Care Network in Brazil (1). These health establishments are important for ensuring continuity of care begun in primary care, ensuring follow-up and improvement of dental treatment offered to the population (2,3).

They should provide services in the clinical areas of oral diagnosis, periodontics, minor oral surgery, endodontics and care for patients with special needs (4,5). The amount of financial incentives received from the Ministry of Health occurs according to the size of the establishment, being classified based on the number of dental treatment rooms: type I (three complete dental treatment rooms), type II (four to six complete dental treatment rooms) and type III (seven or more complete dental treatment rooms) (4,5). 

In order to receive federal funds on a regular basis, certain criteria must be met, including achieving the production targets for procedures, taking into account their size (6). The targets cover the minimum specialties of the Dental Specialty Centers, and failure to achieve them for two consecutive months or three alternating months in the year may result in the suspension of funds until the situation is regularized (6).

The presence of difficulties in achieving the targets set may indicate that the population has difficulty accessing specialized dental care or internal problems (7). For this reason, it is important to analyze the variables that may impact their production and, consequently, achievement of the targets.

At the regional level, the literature has reported association between the unsatisfactory performance of Dental Specialty Centers in achieving the targets and demographic variables, such as a low Municipal Human Development Index (3). However, it is essential to conduct larger-scale studies, covering the whole of Brazil, based on different contextual factors. Understanding these aspects will allow us to uncover weaknesses and reorganize secondary care throughout Brazil (8).

Research related to monitoring the performance of Dental Specialty Centers at regional and local levels with the aim of analyzing associated factors has been carried out (3,7,9,10,11). However, there are few investigations that contemplate the period before and during the COVID-19 pandemic. Studies covering this period are essential in order to reflect the impact that it caused in the area of ​​oral health.

The process of monitoring Dental Specialty Centers should be carried out regularly and the data and information obtained should be considered in planning, choosing priorities and conducting actions in health services (10). Therefore, this study aims to contribute to service management and should result in actions that seek to improve their provision for the entire population, enabling the construction of a care network that allows comprehensive dental care (9).

As such, the objective of this study was to evaluate the performance of the Dental Specialty Centers in Brazil from 2019 to 2022 and analyze its association with contextual factors.

## Methods

### Design

This is an exploratory, observational, cross-sectional, descriptive and analytical study, based on secondary data from the Outpatient Information Systems, obtained via the Information Technology Department of the Brazilian National Health System (DATASUS). The data referred to procedures performed by Dental Specialty Centers between 2019 and 2022.

### Setting

Ministry of Health Ordinance No. 1464/2011 provides for the funding of Dental Specialty Centers. To this end, it establishes that these Centers shall be monitored based on the analysis of minimum monthly dental procedure production (6). 

In this study, dental procedures were consolidated and grouped into six types: basic general procedures, basic restorative procedures, general endodontics, permanent dentition endodontics with three or more roots, periodontics and surgery, as also defined by the Ordinance cited above. Analysis of production was performed considering the groups of dental procedures, according to the targets set for each specialty and by type of Dental Specialty Center (Table 1). Targets were considered to have been met for those specialties that achieved, in the period analyzed, the standardized quantity for each group of specialized dental procedures (6).

**Table 1:** Annual targets, as per Ordinance No. 1464/2011, for the number of procedures performed by Dental Specialty Centers, taking into consideration type of procedure and type of center

Type of procedure	Annual target		
	Dental Specialty Centers type I	Dental Specialty Centers type II	Dental Specialty Centers type III
**Basic** – **general**	960	1,320	2,280
**Basic** – **restorative**	50% of general basic procedures		
**Endodontics** – **general**	420	720	1,140
**Endodontics** – **permanent dentition with three or more roots**	20% of general endodontic procedures		
Periodontics	720	1,080	1,800
Surgery	960	1,080	2,040

### Participants

We evaluated all Dental Specialty Centers in Brazil accredited by the Ministry of Health, which input production data from January 2019 to December 2022 via the Outpatient Information System of the Brazilian National Health System and which were registered on the National Health Establishment Registration System. Centers that were de-accredited by the Ministry of Health or that did not input the production of procedures during the period analyzed were excluded from this research.

### Variables

The number of targets achieved ranged from zero to six, that is, from none to all targets achieved. In order to define the dependent variable, performance was dichotomized into “poor” (0 to 4 targets) and “good” (5 to 6 targets).

The independent variables relating to the municipalities (sociodemographic variables), to the services and to the period (years) were:

- Macro-region: Norte, Northeast, Midwest, Southeast and South.
- Municipal human development index: very low (from 0.000 to 0.499); low (from 0.500 to 0.599); medium (from 0.600 to 0.699); high (from 0.700 to 0.799) and very high (from 0.800 to 1.000). This classification was made according to the United Nations Development Program Atlas of Human Development in Brazilian Metropolitan Regions (*Atlas do desenvolvimento humano nas regiões metropolitanas* brasileiras).
- Municipality population size: 1 (up to 20,000 inhabitants), 2 (from 20,001 to 50,000 inhabitants), 3 (from 50,001 to 100,000 inhabitants) and 4 (more than 100,000 inhabitants).
- Type of Dental Specialty Center: type I (3 dental chairs), type II (4-6 dental chairs) and type III (7 or more dental chairs).
- Type of management: District, Municipal, State and Federal.
- Period (year): 2019, 2020, 2021 and 2022.

### Data sources

In order to obtain information regarding the production achieved by each establishment, the type of dental specialty center (type I, II or III) and type of management (District, Municipal, State or Federal), we used the Brazilian National Health System Outpatient Information System, according to Dental Specialty Centers in all Brazilian regions registered with the National Registry of Health Establishments during the period from 2019 to 2022. 

The contextual data of the municipalities regarding demographic variables, such as Municipal Human Development Index, municipal population size and Brazilian macro-region, were obtained from the Brazilian Institute of Geography and Statistics.

### Bias

Because this is a study using secondary data, strategies were adopted to minimize bias. With this in mind, all records were organized in a standardized way on a spreadsheet and reviewed by more than one of the study’s researchers, with the aim of avoiding inconsistencies and duplication of information.

### Study size

For a national census-type survey, the sample size must correspond to the entire target population. Therefore, information was collected on dental procedure production from all Dental Specialty Centers in Brazil, following the study’s inclusion and exclusion criteria.

### Statistical methods

The production data were tabulated using the DATASUS TabWin application and then exported to Excel, version 2000 (Microsoft Corp.). Initially, the data were analyzed using descriptive statistics to characterize the sample, obtaining the absolute and relative distributions during the four years. Then, a robust Poisson multiple regression model was used to determine the prevalence ratio (PR) of target achievement in the years 2019, 2020, 2021, and 2022. The independent variables were analyzed individually with the outcomes; variables with p-value<0.200 were included in the initial model and those with p-value<0.050 were kept in the adjusted model. A 95% confidence interval (95%CI) was obtained for the variables studied. The data were tabulated and analyzed using JAMOVI (version 2.3.28).

## Results

This study analyzed 1,180 Dental Specialty Centers in 2019; 1,207 in 2020; 1,195 in 2021; and 1,190 in 2022.

**Figure 1:**
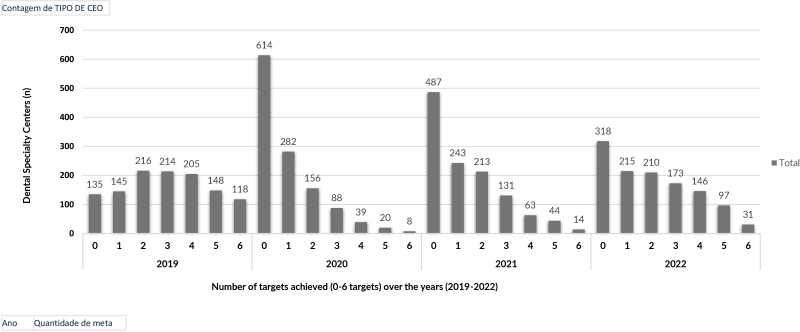
Dental Specialty Centers distribution in relation to the number of targets achieved (0-6 targets). Brazil, 2019-2022 (n=4,773)

Figure 1 shows the number of targets achieved (from 0 to 6) in each year of study. In 2019, most Centers managed to achieve 2 and 3 targets (n=216 and 214, respectively). In turn, in 2020, there was a greater concentration of establishments that did not achieve any target (n=614). In 2021 and 2022, the number of Centers that did not achieve any targets was 487 and 318, respectively. A reduction is observed in the number of centers that did not meet any targets between the years 2021 and 2022.

**Figure 2:**
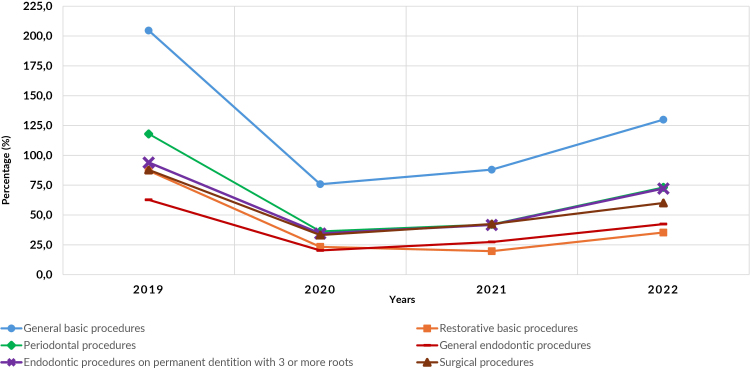
Percentage target achievement per type of dental procedure performed in the Dental Specialty Centers. Brazil, 2019-2022 (n=4,773)

Figure 2 compares the percentage of targets achieved for the six types of procedures individually (general basic procedures, basic restorative procedures, general endodontics, endodontics in permanent teeth with three or more roots, periodontics, and surgery) over the four years of the study. The target achieved for general basic procedures had the best result, while the targets achieved for general endodontics and basic restorative procedures had the lowest results. Even in 2022, with the growth in target achievement compared to the previous two years, no specialty managed to return to the same performance as achieved in 2019 (Figure 2).

Table 2 shows the values ​​referring to the number of Dental Specialty Centers that achieved performance classified as poor (0 to 4 targets achieved) and good (5 to 6 targets achieved). For all municipal variables (macro-region, population size, Municipal Human Development Index), services (type of dental specialty center and type of management) and period (year) of the study, poor performance (0 to 4 targets) prevailed, reaching values ​​above 70%. Concentration of active establishments prevailed in the Northeast region (n=1,903). However, in relation to performance, the Northern region (14.1%) and the Southeast region (13.0%) obtained better results.

**Table 2:** Distribution of Dental Specialty Center performance in achieving goals (0-4 targets: poor performance; 5-6 targets: good performance), according to contextual and temporal variables. Brazil, 2019-2022 (n=4,773)

Variables	Performance	
	Poor n (%)	Good n (%)
Macro-region		
Midwest	315 (90.5)	33 (9.5)
Northeast	1,765 (92.7)	138 (7.3)
Norte	262 (85.9)	43 (14.1)
Southeast	1,434 (87.0)	214 (13.0)
South	517 (90.9)	52 (9.1)
**Municipal Human Development Index**		
Low	626 (93.0)	47 (7.0)
Medium	1,209 (90.4)	128 (9.6)
High	2,100 (89.4)	249 (10.6)
Very high	352 (86.7)	54 (13.3)
Not classified	6 (75.0)	2 (25.0)
**Population size**		
Up to 20,000	670 (92.7)	53 (7.3)
>20,000-50,000	1,193 (92.3)	100 (7.7)
>50,000-100,000	760 (89.2)	92 (10.8)
>100,000	1,670 (87.7)	235 (12.3)
**Type of Dental Specialty Center**		
I	1,868 (91.2)	180 (8.8)
II	1,883 (89.1)	231 (10.9)
III	542 (88.7)	69 (11.3)
**Type of management**		
District	51 (98.1)	1 (1.9)
State	182 (89.2)	22 (10.8)
Federal	18 (94.7)	1 (5.3)
Municipal	4,042 (89.9)	456 (10.1)
**Period** (years)		
2019	915 (77.5)	266 (22.5)
2020	1,179 (97.7)	28 (2.3)
2021	1,137 (95.1)	58 (4.9)
2022	1,062 (89.2)	128 (10.8)

The majority of Dental Specialty Centers are located in municipalities with a high Municipal Human Development Index, and of these, only 10.6% (n=249) managed to achieve 5 or 6 targets. Municipalities with a population size greater than 100,000 inhabitants had better Dental Specialty Center performance (12.4%), achieving 5 to 6 targets, when compared to municipalities with up to 20,000 inhabitants (7.3%). Regarding the type of center, type III had better performance in meeting the targets (11.3%). Most establishments were managed by the municipality (94.2%). However, those under state management had a higher percentage (10.8%) for meeting 5 to 6 targets (good performance). During 2020, only 28 (2.3%) centers managed to achieve 5 to 6 targets.

According to the adjusted Poisson regression model (Table 3), the factors related to greater target achievement (5 to 6 targets) in Dental Specialty Centers were population size and evaluation period (year). Taking into account the PR and 95%CI, we found that centers located in municipalities with more than 100,000 inhabitants presented a higher prevalence of target achievement (PR 1.68; 95%CI 1.26; 2.29) than those found in municipalities with less than 20,000 inhabitants. Regarding the year, the years 2020 (PR 0.10; 95%CI 0.07; 0.15), 2021 (PR 0.22; 95%CI 0.16; 0.28) and 2022 (PR 0.48; 95%CI 0.39; 0.59) target achievement prevalence was lower than in 2019.

**Table 3:** Crude and adjusted prevalence ratios (PR) and 95% confidence intervals (95%CI) for the best performance of the Dental Specialty Centers in achieving targets (5-6 targets) according to study variables. Brazil, 2019-2022 (n=4,773)

Variables	Bivariate		Unadjusted Multivariate^a^		Adjusted Multivariate^b^	
	PR (95%CI)	p-value	PR (95%CI)	p-value	PR (95%CI)	p-value
Macro-region						
South	1.00		1.00			
Southeast	1.42 (1.07; 1.89)	0.862	1.37 (1.04; 1.81)	0.023		
Norte	1.54 (1.06; 2.25)	0.137	1.24 (0.84; 1.84)	0.280		
Northeast	0.79 (0.58; 1.08)	0.025	0.67 (0.46; 0.96)	0.029		
Midwest	1.04 (0.68; 1.57)	0.017	0.98 (0.65; 1.47)	0.916		
**Municipal Human Development Index**						
Low	1.00		1.00			
Medium	1.37 (0.99; 1.89)	0.054	1.02 (0.74; 1.41)	0.910		
High	1.51 (1.12; 2.05)	0.006	0.68 (0.45; 1.02)	0.063		
Very high	1.90 (1.31; 2.76)	<0.001	0.73 (0.45; 1.21)	0.223		
**Population size**						
0-20,000	1.00		1.00		1.00	
20,001-50,000	1.05 (0.77; 1.45)	0.743	1.01 (0.74; 1.37)	0.940	1.05 (0.76; 1.48)	0.768
50,001-100,000	1.47 (1.07; 2.03)	0.019	1.44 (1.05; 1.98)	0.024	1.47 (1.05; 2.07)	0.026
>100,000	1.68 (1.26; 2.23)	<0.001	1.62 (1.19; 2.20)	0.002	1.68 (1.26; 2.29)	<0.001
**Type of Dental Specialty Center**						
I	1.00					
II	1.24 (1.03; 1.50)	0.021				
III	1.28 (0.99; 1.67)	0.061				
**Type de management**						
Municipal	1.00					
Federal	0.51 (0.08; 3.50)	0.094				
State	1.06 (0.71; 1.59)	0.764				
District	0.19 (0.03; 1.32)	0.501				
**Period** (year)						
2019	1.00				1.00	
2020	0.10 (0.07; 0.15)	<0.001	0.10 (0.07; 0.15)	<0.001	0.10 (0.07; 0.15)	<0.001
2021	0.21 (0.16; 0.28)	<0.001	0.22 (0.16; 0.28)	<0.001	0.22 (0.16; 0.28)	<0.001
2022	0.48 (0.39; 0.58)	<0.001	0.47 (0.39; 0.57)	<0.001	0.48 (0.39; 0.59)	<0.001

^a^Initial multivariate analysis model, incorporating variables with p-value<0.200; ^b^Adjusted multivariate analysis model, incorporating variables with p-value<0.050.

## Discussion

During the four years analyzed, only a minority of Dental Specialty Centers managed to achieve all procedure targets. General endodontics and basic restorative procedures had the lowest target achievements. Low compliance with establishment targets was seen especially in the years 2020 and 2021 and in municipalities with smaller populations.

A limitation of this study is the possibility of errors in the recording of information on databases and limited availability of comparative studies that analyze the performance of Dental Specialty Centers throughout Brazil. It is worth noting that this research presents representative data for Brazil and, therefore, this limitation is not particularly detrimental to the evaluation. Due to the limited availability of indicator databases, the difficulty of including other variables for in-depth analysis is another limitation of this study. For this reason, the future prospect of conducting new research with the inclusion of new variables, such as the number of specialist hired per municipality, is essential for further exploration of the topic.

Different factors may have influenced the lower achievement of targets during years 2020 and 2021. Considering that the COVID-19 pandemic was declared by the World Health Organization in 2020 (12), it can be understood that, from that period onwards, changes in the health sector occurred in order to adapt to the new adversities.

Among the measures that could be taken, the Ministry of Health implemented instructions only regarding the provision of services classified as urgent and emergency, so at to avoid unnecessary exposure of health professionals (13,14). This attitude was justified in the context of oral health, since dental treatment rooms are environments with a high risk of infection for professionals and patients, due to direct exposure to COVID aerosols during care (15). 

The population’s reluctance to use health services due to fear of contracting the virus was another factor observed (16). A study that sought to assess the impact of COVID-19 on the provision of dental procedures in the Brazilian National Health System found that, when comparing the years 2019 and 2020, there was a reduction of up to 92.3% in consultations and non-emergency dental care in specialized care (16). The problem with this is that, often, difficulty in accessing health care leads to uncontrolled self-medication, lack of instructions and delay in early diagnosis of lesions (18-20).

The decrease in primary care services during this period may also be a hypothesis for the decrease in secondary care services, which would consequently contribute to lower dental procedure production. This is because the referral flow generated from primary care to secondary care is considered an important gateway for users to arrive at Dental Specialty Centers. A study that sought to evaluate dental care in primary care by means of an epidemiological survey from 2019 to 2022, indicated a 50.4% decrease in dental care services in primary care from 2019 to 2020, that is, before and during the pandemic, respectively (21).

Given this context, it is our understanding that the typical form of monitoring Dental Specialty Centers does not fully adapt to adverse conditions. For this reason, there is a need to create monitoring models that can be adapted to atypical situations, such as health emergency problems.

As an adaptation measure in light of the global context, Technical Note No. 4/2022-General Coordination of Oral Health/Department of Family Health/Secretariat of Primary Health Care/Ministry of Health was prepared with the aim of guaranteeing financial transfers to dental care establishments, regardless of whether monthly production targets were reached up to February 2022 (22). This measure to maintain receipt of funding can be interpreted not as a failure in the production model, but rather as recognition of the essential nature of the service for the public health network.

Analyzing each target individually, it can be seen that general basic procedures achieved the best performance. As a possible hypothesis for these procedures having the best result in achieving targets, it should be considered that Ordinance No. 1464/2011 defined that in the case of patients with special needs, these procedures should be performed exclusively in Dental Specialty Centers (6). Patients with impeding disabilities require greater dental care and attention and, therefore, secondary care promotes the concentration of basic procedures performed on these users, through trained dentists. It should also be taken into account that, during the pandemic period, the care provided to these users was categorized as essential elective care and, therefore, that oral health care for this group of people should not be postponed (23).

During the four years, general basic procedures obtained the best results, while the percentage of general endodontics procedures and basic restorative procedures concentrated the lowest results. A study carried out in the state of Minas Gerais with the aim of analyzing secondary health care found that 77.0% of the municipalities in Minas Gerais did not perform any endodontic procedures (11). Lack of endodontic procedures can be affected by a series of issues that make performing them difficult, such as, for example, the arrival of patients at the Dental Specialty Center with accumulated bacterial plaque, low availability of endodontics professional working hours, service protocol, work process, problems with equipment, among others.

It was also found that general endodontic procedures had poorer results when compared to endodontic procedures for permanent teeth with three or more roots. This result differs from that reported by other studies (23,24), which found that endodontic procedures were mostly performed on single- or double-rooted permanent teeth, with premolars coming first, followed by mandibular molars.

Our study showed the presence of association between population size and Dental Specialty Centers meeting 5 and 6 targets. Thus, municipalities with a population size above 100,000 inhabitants achieved better results, to the detriment of municipalities with smaller population sizes. This response was explained in a survey carried out in Belo Horizonte in 2020, which analyzed extraction of permanent teeth and found that municipalities with higher population density also have a high demand and, consequently, the number of treatments performed tends to increase proportionally (25).

In this way, this study makes it possible to identify weaknesses and create new plans designed by health service management to improve results regarding meeting targets. It is also important that, with the findings obtained in this research, there be discussion on the creation of new tools and the use of more comprehensive performance indicators that take into account not only quantitative information, but also the ability of health services to adapt to disadvantageous scenarios.

Based on this research, we conclude that most Dental Specialty Centers performed poorly in terms of achieving their specialty targets. Municipalities with larger population sizes were more likely to achieve their targets, and the COVID-19 pandemic (2020 to 2022) negatively influenced the performance of these establishments.

## Data Availability

The secondary data collected for this research are available at: https://datasus.saude.gov.br/informacoes-de-saude-tabnet/
