# Supplementary material for: Analysis of Dental Specialty Center target achievement and associated factors, Brazil, 2019-2022: a cross-sectional study
Source: Epidemiol Serv Saude. 2025 Jul 11;34:e20240610. doi: 10.1590/S2237-96222025v34e20240610.en (PMC12266655; doi:10.1590/S2237-96222025v34e20240610.en)
Supplement: Translation

# Translation: Análise do cumprimento de metas dos Centros de Especialidades Odontológicas e fatores associados, Brasil, 2019-2022: estudo transversal

**DOI:** 10.1590/S2237-96222025v34e20240610.pt

**Authors:** Luciana Leônia Soares Freire, Rênnis Oliveira da Silva, Rilary Rodrigues Feitosa, Lucas Xavier Bezerra de Menezes, Renato Taqueo Placeres Ishigame, Yuri Wanderley Cavalcanti, Edson Hilan Gomes de Lucena

**Affiliations:** 1 Universidade Federal da Paraíba, Programa de Pós-Graduação em Odontologia, João Pessoa, PB, Brasil; 2 Universidade Federal da Paraíba, Curso de Odontologia, João Pessoa, PB, Brasil; 3 Ministério da Saúde, Coordenação-Geral de Saúde Bucal, Brasília, DF, Brasil; 4 Universidade Federal da Paraíba, Departamento de Clínica e Odontologia Social, João Pessoa, PB, Brasil

**Keywords:** Saúde Bucal, Atenção Secundária à Saúde, Especialidades Odontológicas, Covid-19, Avaliação em Saúde

## Abstract

**Objetivo:**

Avaliar o desempenho dos Centros de Especialidades Odontológicas do Brasil, de 2019 a 2022, e analisar sua associação com fatores contextuais.

**Métodos:**

Trata-se de um estudo observacional, transversal, descritivo e analítico. Foram utilizados dados secundários da produção dos Centros de Especialidades Odontológicas coletados do Sistemas de Informações Ambulatoriais. Um modelo robusto de regressão múltipla de Poisson foi obtido para determinar a razão de prevalência (RP) do alcance das metas. O intervalo de confiança de 95% (IC95%) foi obtido para as variáveis em estudo.

**Resultados:**

O desempenho ruim, com o alcance de 0 a 3 metas, prevaleceu nos Centros de Especialidades Odontológicas. Entre os procedimentos, as metas referentes aos Procedimentos básicos gerais tiveram o melhor resultado, com o maior cumprimento de metas. Centros de Especialidades Odontológicas localizados em regiões com mais de 100 mil habitantes apresentaram maior frequência de alcance de meta (RP 1,68; IC95% 1,26; 2,29). Os anos de 2020 (RP 0,10; IC95% 0,07; 0,15), 2021 (RP 0,22; IC95% 0,16; 0,28) e 2022 (RP 0,48; IC95% 0,39; 0,59) apresentaram menor prevalência de alcance de metas que 2019.

**Conclusão:**

Grande parte dos Centros de Especialidades Odontológicas apresentaram desempenho ruim quanto ao alcance das metas. Municípios de maior porte populacional tiveram maior probabilidade de alcançar as metas. A pandemia de covid-19 influenciou negativamente no desempenho.

**Aspectos éticos**

Esta pesquisa utilizou bancos de dados de domínio público e anonimizados.:

## Introdução

Os Centros de Especialidades Odontológicas são responsáveis por oferecer serviços de saúde bucal especializados, integrando-se como um importante componente da Rede de Atenção em Saúde Bucal no Brasil (1). Esses estabelecimentos de saúde são importantes para assegurar a continuidade do cuidado iniciado na atenção básica, garantindo o seguimento e o aprimoramento do tratamento odontológico oferecido à população (2,3).

Eles devem ofertar atendimentos nas áreas clínicas de diagnóstico bucal, periodontia, cirurgia oral menor, endodontia e atendimento a pacientes com necessidades especiais (4,5). O recebimento de incentivo financeiro do Ministério da Saúde ocorre de acordo com o porte do estabelecimento, sendo classificado a partir da quantidade de consultórios odontológicos: tipo I (três consultórios odontológicos completos), tipo II (quatro a seis consultórios odontológicos completos) e tipo III (sete ou mais consultórios odontológicos completos) (4,5). 

Para o recebimento regular dos recursos federais, é necessário o cumprimento de alguns critérios, entre eles, o alcance das metas de produção de procedimentos, considerando-se o porte (6). As metas abrangem as especialidades mínimas dos Centros de Especialidades Odontológicas, e o não alcance por dois meses consecutivos ou três meses alternados no ano pode implicar a suspensão dos recursos, até a regularização da situação (6). 

A presença de dificuldades no alcance das metas estabelecidas pode indicar uma dificuldade de acesso da população aos atendimentos odontológicos especializados ou problemas internos (7). Por esse motivo, é importante que sejam analisadas as variáveis que podem impactar na sua produção e, consequentemente, no alcance das metas. 

Observou-se na literatura, em nível regional, a associação do desempenho insatisfatório do alcance das metas dos Centros de Especialidades Odontológicas com variáveis demográficas, como o baixo Índice de Desenvolvimento Humano Municipal (3). No entanto, é fundamental a realização de estudos em maior escala, abrangendo todo o Brasil, a partir de diferentes fatores contextuais. Conhecer esses aspectos permite desvendar as fragilidades e reorganizar a atenção secundária em todo o território brasileiro (8).

Pesquisas relacionadas ao monitoramento do desempenho dos Centros de Especialidades Odontológicas em âmbito regional e local com intuito de analisar os fatores associados foram realizadas (3,7,9,10,11). Entretanto, são escassas investigações que contemplem o período antes e durante da pandemia do covid-19. Estudos abarcando esse período são indispensáveis para refletir o impacto que o momento gerou na área da saúde bucal. 

O processo de monitoramento dos Centros de Especialidades Odontológicas deve ser realizado de forma regular e os dados e as informações devem ser considerados no planejamento, escolhas de prioridades e condução de ações nos serviços de saúde (10). Sendo assim, esse trabalho visa contribuir com a gestão dos serviços e deve resultar em ações que busquem melhorar a oferta para toda a população, possibilitando a construção de uma rede de atenção que permita cuidado odontológico integral (9).

A partir disso, o presente trabalho teve como objetivo avaliar o desempenho dos Centros de Especialidades Odontológicas do Brasil, de 2019 a 2022, e analisar sua associação com fatores contextuais.

## Métodos

### Desenho do estudo

Trata-se de estudo exploratório do tipo observacional, transversal, descritivo e analítico, a partir de dados secundários dos Sistemas de Informações Ambulatoriais, obtidos por meio do Departamento de Informática do Sistema Único de Saúde (DATASUS). Os dados foram referentes à produção de procedimentos realizados, por centro de especialidades odontológicas, durante o período de 2019 a 2022.

### Contexto

A Portaria do Ministério da Saúde nº 1.464/2011 contempla o financiamento dos Centros de Especialidades Odontológicas. Para isso, estabelece o monitoramento desses centros com base na análise da produção mínima mensal de procedimentos odontológicos (6). 

Nesta pesquisa, os procedimentos odontológicos foram consolidados e agrupados em seis tipos: procedimentos básicos gerais, básicos restauradores, de endodontia geral, de endodontia em dentes permanentes com três raízes ou mais, de periodontia e de cirurgia, conforme definido pela mesma portaria. A análise da produção foi realizada considerando os grupos de procedimentos odontológicos, em função das metas estabelecidas para cada especialidade e por tipo de centro de especialidades odontológicas (Tabela 1). A meta atingida foi considerada para aquelas especialidades que alcançaram, no período analisado, a quantidade normatizada para cada grupo de procedimentos odontológicos especializados (6). 

**Tabela 1:** Metas anuais, de acordo com a Portaria n^o^ 1.464/2011, para a quantidade de procedimentos realizados por Centros de Especialidades Odontológicas, levando em consideração o tipo de procedimento e tipo de centro

Tipo de procedimento	Meta anual		
	Centros de Especialidades Odontológicas tipo I	Centros de Especialidades Odontológicas tipo II	Centros de Especialidades Odontológicas tipo III
**Básicos gerais**	960	1.320	2.280
**Básicos restauradores**	50% dos procedimentos básicos gerais		
**Endodontia geral**	420	720	1.140
**Endodontia em permanentes com três ou mais raízes**	20% dos procedimentos endodônticos gerais		
Periodontia	720	1.080	1.800
Cirurgia	960	1.080	2.040

### Participantes

Foram avaliados todos os Centros de Especialidades Odontológicas do Brasil habilitados pelo Ministério da Saúde, que enviaram dados de produção de janeiro de 2019 a dezembro de 2022 através do Sistema de Informações Ambulatoriais do Sistema Único de Saúde e que estavam registrados no Sistema Nacional de Cadastros de Estabelecimentos de Saúde. Foram excluídos da pesquisa os centros que foram descredenciados pelo Ministério da Saúde ou que não enviaram a produção de procedimentos durante o período analisado.

### Variáveis

A quantidade de metas alcançadas variou de zero a seis, isto é, de nenhuma até todas as metas alcançadas. Para definição da variável dependente, dicotomizou-se o desempenho em “ruim” (de 0 a 4 metas) e “bom” (de 5 a 6 metas).

As variáveis independentes relacionadas aos municípios (sociodemográficas), aos serviços e o período (anos) utilizadas foram:

- Macrorregião: Norte, Nordeste, Centro-Oeste, Sudeste e Sul.
- Índice de Desenvolvimento Humano Municipal: muito baixo (de 0,000 e 0,499>); baixo (de 0,500 a 0,599); médio (de 0,600 a 0,699); alto (de 0,700 a 0,799) e muito alto (de 0,800 a 1,000).Tal classificação foi realizada de acordo com o *Atlas do desenvolvimento humano nas regiões metropolitanas* brasileiras do Programa das Nações Unidas para o Desenvolvimento.
- Porte populacional dos municípios: 1 (até 20.000 habitantes), 2 (de 20.001 a 50.000 habitantes), 3 (de 50.001 a 100.000 habitantes) e 4 (acima de 100.000 habitantes).
- Tipo de centro de especialidades odontológicas: tipo I (3 cadeiras odontológicas), tipo II (4 a 6 cadeiras odontológicas) e tipo III (acima de 7 cadeiras odontológicas).
- Tipo de gestão: Distrital, Municipal, Estadual e Federal.
- Período (ano): 2019, 2020, 2021 e 2022.

### Fontes de dados

Para as informações referentes à produção realizada por cada estabelecimento, o tipo de centro de especialidades odontológicas (tipo I, II ou III) e tipo de gestão (Distrital, Municipal, Estadual ou Federal), utilizou-se o Sistema de Informação Ambulatorial do Sistema Único de Saúde, segundo o Cadastro Nacional de Estabelecimento de Saúde dos Centros de Especialidades Odontológicas de todas as regiões brasileiras durante o período de 2019 a 2022. 

Os dados contextuais dos municípios referentes às variáveis demográficas, como índice de desenvolvimento humano municipal, porte populacional dos municípios e macrorregião brasileira, foram obtidos do Instituto Brasileiro de Geografia e Estatística. 

### Viés

Por se tratar de uma pesquisa com dados secundários, estratégias foram adotadas com intuito de minimizar vieses no estudo. Pensando nisso, todos os registros foram organizados de forma padronizada em uma planilha e revisados por mais de um pesquisador do estudo, com o objetivo de evitar inconsistências e duplicidades de informações. 

### Tamanho do estudo

Para uma pesquisa em nível nacional tipo censo, o tamanho amostral deve corresponder à totalidade da população-alvo. Dessa forma, foram coletadas informações da produção de procedimentos odontológicos de todos os Centros de Especialidades Odontológicas do Brasil, seguindo os critérios de inclusão e exclusão do estudo.

### Métodos estatísticos

Os dados da produção foram tabulados com utilização do programa TabWin do DATASUS, e em seguida exportados para o programa Excel, versão 2000 (Microsoft Corp.). Inicialmente, os dados foram analisados por estatística descritiva para caracterizar a amostra, sendo obtidas as distribuições absolutas e relativas, durante os quatro anos. Em seguida, um modelo robusto de regressão múltipla de Poisson foi realizado para determinar a razão de prevalência (RP) do alcance das metas nos anos 2019, 2020, 2021 e 2022. As variáveis independentes foram analisadas individualmente com os desfechos, as variáveis com p-valor<0,200 foram incluídas no modelo inicial e permaneceram no modelo ajustado aquelas com p-valor<0,050. O intervalo de confiança de 95% (IC95%) foi obtido para as variáveis em estudo. Os dados foram tabulados e analisados no software JAMOVI (versão 2.3.28).

## Resultados

No estudo, foram analisados 1.180 Centros de Especialidades Odontológicas no ano de 2019; 1.207 em 2020; 1.195 em 2021; e 1.190 em 2022.

A Figura 1 mostra a quantidade de metas alcançadas (de 0 a 6) em cada ano de estudo. No ano de 2019, a maior parte dos centros conseguiram atingir 2 e 3 metas (n=216 e 214, respectivamente). Por sua vez, no ano de 2020, houve maior concentração de estabelecimentos que não atingiu nenhuma meta (n=614). Nos anos de 2021 e 2022, a quantidade de centros que não atingiram nenhuma meta correspondeu a 487 e 318, respectivamente. Observa-se uma redução no número de centros que não atingiram nenhuma meta entre os anos de 2021 e 2022.

**Figura 1:** Distribuição dos Centros de Especialidades Odontológicas em relação à quantidade de metas alcançadas (0 a 6 metas). Brasil, 2019-2022 (n=4.773)

A Figura 2 estabelece a comparação do percentual de alcance de metas dos seis tipos de procedimentos individualmente (procedimentos básicos gerais, básicos restauradores, endodontia geral, endodontia em permanentes com três raízes ou mais, periodontia e cirurgia), ao longo dos quatro anos do estudo. O alcance da meta referente aos procedimentos básicos gerais obteve o melhor resultado, enquanto no alcance das metas referentes aos procedimentos de endodontia geral e procedimentos básicos restauradores se concentraram os resultados inferiores. Mesmo em 2022, com o crescimento do alcance das metas, em comparação aos dois anos anteriores, nenhuma especialidade conseguiu retomar o mesmo desempenho de 2019 (Figura 2). 

**Figura 2:** Percentual do alcance de metas por tipo de procedimento odontológico executado nos Centros de Especialidades Odontológicas. Brasil, 2019-2022 (n=4.773)

A Tabela 2 mostra os valores referentes à quantidade de Centros de Especialidades Odontológicas que atingiram desempenho classificado como ruim (0 a 4 metas alcançadas) e bom (5 a 6 metas alcançadas). Em todas as variáveis municipais (macrorregião, porte populacional, Índice de Desenvolvimento Humano Municipal), de serviços (tipo de centro de especialidades odontológicas e tipo de gestão) e período (ano) do estudo, o desempenho ruim (0 a 4 metas) prevaleceu, chegando a valores superiores a 70%. A concentração de estabelecimentos ativos prevaleceu na região Nordeste (n=1.903). Porém, em relação ao desempenho, as regiões Norte (14,1%) e Sudeste (13,0%) apresentaram resultados superiores. 

**Tabela 2:** Distribuição do desempenho dos Centros de Especialidades Odontológicas no alcance de metas (0-4 metas: desempenho ruim; 5-6 metas: desempenho bom), segundo variáveis contextuais e temporais. Brasil, 2019-2022 (n=4.773)

Variáveis	Desempenho	
	Ruim n (%)	Bom n (%)
Macrorregião		
Centro-Oeste	315 (90,5)	33 (9,5)
Nordeste	1.765 (92,7)	138 (7,3)
Norte	262 (85,9)	43 (14,1)
Sudeste	1.434 (87,0)	214 (13,0)
Sul	517 (90,9)	52 (9,1)
**Índice de desenvolvimento humano municipal**		
Baixo	626 (93,0)	47 (7,0)
Médio	1.209 (90,4)	128 (9,6)
Alto	2.100 (89,4)	249 (10,6)
Muito alto	352 (86,7)	54 (13,3)
Sem classificação	6 (75,0)	2 (25,0)
**Porte populacional**		
Até 20.000	670 (92,7)	53 (7,3)
>20.000-50.000	1.193 (92,3)	100 (7,7)
>50.000-100.000	760 (89,2)	92 (10,8)
>100.000	1.670 (87,7)	235 (12,3)
**Tipo de Centro de Especialidades Odontológicas**		
I	1.868 (91,2)	180 (8,8)
II	1.883 (89,1)	231 (10,9)
III	542 (88,7)	69 (11,3)
**Tipo de gestão**		
Distrital	51 (98,1)	1 (1,9)
Estadual	182 (89,2)	22 (10,8)
Federal	18 (94,7)	1 (5,3)
Municipal	4.042 (89,9)	456 (10,1)
**Período** (anos)		
2019	915 (77,5)	266 (22,5)
2020	1.179 (97,7)	28 (2,3)
2021	1.137 (95,1)	58 (4,9)
2022	1.062 (89,2)	128 (10,8)

A maioria dos Centros de Especialidades Odontológicas encontra-se em municípios com Índice de Desenvolvimento Humano Municipal alto e, destes, apenas 10,6% (n=249) conseguiram atingir 5 ou 6 metas. Os municípios com porte populacional maior que 100 mil habitantes obtiveram melhor desempenho de Centros de Especialidades Odontológicas (12,4%), alcançando de 5 a 6 metas, quando comparados a municípios de até 20 mil habitantes (7,3%). Em relação ao tipo de centro, o tipo III possuiu melhor desempenho no alcance das metas (11,3%). A maior parte dos estabelecimentos apresentou gestão municipal (94,2%). Porém, os que estavam sob gestão estadual apresentaram um maior percentual de alcance de 5 a 6 metas (desempenho bom), representado por 10,8%. Durante o ano de 2020, apenas 28 (2,3%) centros conseguiram atingir de 5 a 6 metas.

De acordo com o modelo ajustado de regressão de Poisson (Tabela 3), os fatores relacionados ao maior alcance de metas (5 a 6 metas) nos Centros de Especialidades Odontológicas foram o porte populacional e o período (ano) de avaliação. Levando em consideração a RP e o IC95%, observou-se que os centros localizados em municípios com mais de 100 mil habitantes apresentaram maior prevalência de alcance de metas (RP 1,68; IC95% 1,26; 2,29) que os encontrados em municípios com menos de 20 mil habitantes. Em relação ao ano, os anos de 2020 (RP 0,10; IC95% 0,07; 0,^15^), 2021 (RP 0,22; IC95% 0,16; 0,28) e 2022 (RP 0,48; IC95% 0,39; 0,59) apresentaram menor prevalência de alcance de metas do que o ano de 2019.

**Tabela 3:** Razões de prevalências (RP) brutas e ajustadas e intervalos de confiança de 95% (IC95%) do melhor desempenho dos Centros de Especialidades Odontológicas no alcance de metas (5-6 metas) pelas variáveis do estudo. Brasil, 2019-2022 (n=4.773)

Variáveis	Bivariada		Multivariada Não ajustada^a^		Multivariada Ajustada^b^	
	RP (IC95%)	p-valor	RP (IC95%)	p-valor	RP (IC95%)	p-valor
Macrorregião						
Sul	1,00		1,00			
Sudeste	1,42 (1,07; 1,89)	0,862	1,37 (1,04; 1,81)	0,023		
Norte	1,54 (1,06; 2,25)	0,137	1,24 (0,84; 1,84)	0,280		
Nordeste	0,79 (0,58; 1,08)	0,025	0,67 (0,46; 0,96)	0,029		
Centro-Oeste	1,04 (0,68; 1,57)	0,017	0,98 (0,65; 1,47)	0,916		
**Índice de desenvolvimento humano municipal**						
Baixo	1,00		1,00			
Médio	1,37 (0,99; 1,89)	0,054	1,02 (0,74; 1,41)	0,910		
Alto	1,51 (1,12; 2,05)	0,006	0,68 (0,45; 1,02)	0,063		
Muito alto	1,90 (1,31; 2,76)	<0,001	0,73 (0,45; 1,21)	0,223		
**Porte populacional**						
0-20.000	1,00		1,00		1,00	
20.001-50.000	1,05 (0,77; 1,45)	0,743	1,01 (0,74; 1,37)	0,940	1,05 (0,76; 1,48)	0,768
50.001-100.000	1,47 (1,07; 2,03)	0,019	1,44 (1,05; 1,98)	0,024	1,47 (1,05; 2,07)	0,026
>100.000	1,68 (1,26; 2,23)	<0,001	1,62 (1,19; 2,20)	0,002	1,68 (1,26; 2,29)	<0,001
**Tipo de Centro de Especialidades Odontológicas**						
I	1,00					
II	1,24 (1,03; 1,50)	0,021				
III	1,28 (0,99; 1,67)	0,061				
**Tipo de gestão**						
Municipal	1,00					
Federal	0,51 (0,08; 3,50)	0,094				
Estadual	1,06 (0,71; 1,59)	0,764				
Distrital	0,19 (0,03; 1,32)	0,501				
**Período** (ano)						
2019	1,00				1,00	
2020	0,10 (0,07; 0,15)	<0,001	0,10 (0,07; 0,15)	<0,001	0,10 (0,07; 0,15)	<0,001
2021	0,21 (0,16; 0,28)	<0,001	0,22 (0,16; 0,28)	<0,001	0,22 (0,16; 0,28)	<0,001
2022	0,48 (0,39; 0,58)	<0,001	0,47 (0,39; 0,57)	<0,001	0,48 (0,39; 0,59)	<0,001

^a^Modelo inicial da análise multivariada, incorporadas variáveis com p-valor<0,200; ^b^Modelo ajustado da análise multivariada, incorporadas variáveis com p-valor<0,050.

## Discussão

Durante os quatro anos analisados, uma minoria de Centros de Especialidades Odontológicas conseguiu atingir todas as metas de procedimentos. Os procedimentos de endodontia geral e básicos restauradores tiveram os menores alcances de metas. O baixo cumprimento das metas dos estabelecimentos foi observado especialmente nos anos de 2020 e 2021 e nos municípios com menor população. 

O estudo traz como limitação a possibilidade de erros no registro das informações pelos bancos de dados e restrições na disponibilidade de estudos comparativos que analisem o desempenho dos Centros de Especialidades Odontológicas em todo o território brasileiro. Cabe salientar que esta pesquisa traz dados representativos do Brasil e, dessa forma, essa limitação não implica tantos prejuízos na avaliação. Devido à disponibilidade restrita dos bancos de dados de indicadores, a dificuldade de incluir outras variáveis para aprofundamento da análise é mais uma limitação deste estudo. Por esse motivo, a perspectiva futura de realizar novas pesquisas com inclusão de novas variáveis, como o número de contratações de especialistas por municípios, é essencial para o aprofundamento do tema.

Diferentes fatores podem ter influenciado no menor alcance de metas durante os anos de 2020 e 2021. Considerando-se que a pandemia de covid-19 foi decretada pela Organização Mundial da Saúde em 2020 (12), é possível compreender que, a partir desse período, mudanças no âmbito da saúde ocorreram para adaptação às novas adversidades. 

Entre as medidas cabíveis, o Ministério da Saúde implementou instruções apenas quanto à prestação de atendimentos enquadrados como urgência e emergência, evitando a exposição desnecessária de profissionais da saúde (13,14). Essa atitude foi justificada no âmbito da saúde bucal, uma vez que o consultório odontológico é um ambiente com alto risco de infecção para os profissionais e pacientes, devido à exposição direta a aerossóis durante o atendimento (15). 

A resistência da população em frequentar os serviços de saúde por causa do medo de contágio pelo vírus também foi outro fator observado (16). Um estudo buscou avaliar o impacto do covid-19 na oferta de procedimentos odontológicos no Sistema Único de Saúde e observou que, ao serem comparados os anos de 2019 e 2020, houve uma redução de até 92,3% das consultas e dos atendimentos odontológicos não emergenciais na atenção especializada (16). O problema disso é que, muitas vezes, a dificuldade de acesso à saúde leva a automedicação descontrolada, falta de instruções e demora no diagnóstico precoce de lesões (18-20).

A diminuição dos atendimentos na atenção básica durante esse período também pode ser uma hipótese da diminuição de atendimentos na atenção secundária, o que consequentemente contribuiria para a menor produção de procedimentos. Isso porque o fluxo de referência gerado da atenção básica para a atenção secundária é considerado uma importante porta de entrada para a chegada de usuários aos Centros de Especialidades Odontológicas. Uma pesquisa buscou avaliar o atendimento odontológico na atenção primária através de um levantamento epidemiológico de 2019 a 2022, e apontou um decréscimo de 50,4% de atendimentos odontológicos na atenção primária de 2019 para 2020, ou seja, antes e durante a pandemia, respectivamente (21).

Diante desse contexto, compreende-se que o monitoramento dos Centros de Especialidades Odontológicas realizado na sua forma típica não se adapta totalmente a condições adversas. Por esse motivo, existe a necessidade da criação de modelos de monitoramento adaptáveis a situações atípicas, como problemas de emergências sanitárias. 

Como medida de adaptação frente ao contexto mundial, a Nota Técnica nº 4/2022-Coordenação Geral de Saúde Bucal/Departamento de Saúde da Família/Secretaria de Atenção Primária à Saúde/Ministério da Saúde foi proposta com intuito de garantir o repasse financeiro aos estabelecimentos, independentemente do alcance de metas mensais de produção até o mês fevereiro de 2022 (22). Essa medida de manutenção do recebimento financeiro pode ser interpretada não como uma falha no modelo de produção, mas sim como reconhecimento da essencialidade do serviço para a rede pública de saúde.

Analisando-se individualmente cada meta, verificou-se que os procedimentos básicos gerais obtiveram o melhor desempenho. Como uma possível hipótese para o melhor resultado de alcance de metas no caso de procedimentos básicos gerais, deve-se considerar que a Portaria nº 1.464/2011 definiu exclusividade desses procedimentos nos Centros de Especialidades Odontológicas para pacientes com necessidades especiais (6). Os pacientes com deficiências impeditivas carecem de maior cuidado e atenção odontológica e, por isso, a atenção secundária promove a concentração de realizações de procedimentos básicos nesses usuários, através de cirurgiões-dentistas capacitados. Também se deve levar em consideração que, durante o período de pandemia, os atendimentos realizados nesses usuários foram categorizados como eletivos essenciais e, dessa forma, a atenção à saúde bucal desse grupo de pessoas não deveria ser adiada (23). 

Durante os quatro anos, os procedimentos básicos gerais obtiveram o melhor resultado, enquanto o percentual de procedimentos de endodontia geral e procedimentos básicos restauradores concentraram os resultados mais baixos. Um estudo realizado em Minas Gerais com intuito de analisar a assistência secundária em saúde observou que 77,0% dos municípios mineiros não realizaram nenhum procedimento endodôntico (11). A falta de procedimentos endodônticos pode ser afetada por uma série de questões que dificultam a sua execução, como, por exemplo, a chegada de pacientes ao centro de especialidades odontológicas com um meio bucal inadequado, baixa oferta de carga horária dos profissionais de endodontia, protocolo de atendimento, processo de trabalho, dificuldade de equipamentos, entre outros. 

Também foi observado que os procedimentos de endodontia geral tiveram resultados inferiores quando comparados aos procedimentos de endodontia em permanentes com três raízes ou mais. Esse resultado difere do exposto por outras pesquisas (23,24), que observaram a realização de procedimentos endodônticos em sua maior parte feita em dentes permanentes uni ou birradiculares, sendo em primeiro lugar os pré-molares, seguidos de molares inferiores.

A pesquisa mostrou a presença de associação entre o porte populacional e o cumprimento de 5 e 6 metas pelos Centros de Especialidades Odontológicas. Desse modo, municípios com porte populacional acima de 100 mil habitantes atingiram melhores resultados, em detrimento de municípios de menor porte populacional. Essa resposta foi justificada em uma pesquisa realizada em Belo Horizonte no ano de 2020, que analisou as exodontias de dentes permanentes e observou que municípios com densidade demográfica maior apresentam também uma elevada demanda e, por consequência, os números de tratamentos realizados tendem a crescer proporcionalmente (25). 

Dessa maneira, a realização deste estudo possibilita a identificação das fragilidades e a construção de novos planos traçados pela gestão voltados para melhoria nos resultados do cumprimento de metas. Também é importante que, com os achados obtidos nesta pesquisa, se abra a discussão sobre a criação de novas ferramentas e o uso de indicadores de desempenho mais abrangentes que levem em consideração não apenas informações quantitativas, mas também a capacidade de adaptação dos serviços de saúde frente a cenários desvantajosos. 

Conclui-se, a partir desta pesquisa, que a maior parte dos Centros de Especialidades Odontológicas apresentaram desempenho ruim no que diz respeito ao alcance das metas das especialidades. Os municípios de maior porte populacional tiveram maior probabilidade de alcançar as metas e a pandemia de covid-19 (2020 a 2022) influenciou negativamente no desempenho desses estabelecimentos.

## Data Availability

Os dados secundários coletados para a pesquisa estão disponíveis em: https://datasus.saude.gov.br/informacoes-de-saude-tabnet/
